# Mechanisms of Polarized Organelle Distribution in Neurons

**DOI:** 10.3389/fncel.2016.00088

**Published:** 2016-03-31

**Authors:** Dylan J. Britt, Ginny G. Farías, Carlos M. Guardia, Juan S. Bonifacino

**Affiliations:** Cell Biology and Neurobiology Branch, Eunice Kennedy Shriver National Institute of Child Health and Human Development, National Institutes of HealthBethesda, MD, USA

**Keywords:** neurons, polarized sorting, organelle distribution, axon initial segment, pre-axonal exclusion zone, microtubules, kinesins, axonal transport

## Abstract

Neurons are highly polarized cells exhibiting axonal and somatodendritic domains with distinct complements of cytoplasmic organelles. Although some organelles are widely distributed throughout the neuronal cytoplasm, others are segregated to either the axonal or somatodendritic domains. Recent findings show that organelle segregation is largely established at a pre-axonal exclusion zone (PAEZ) within the axon hillock. Polarized sorting of cytoplasmic organelles at the PAEZ is proposed to depend mainly on their selective association with different microtubule motors and, in turn, with distinct microtubule arrays. Somatodendritic organelles that escape sorting at the PAEZ can be subsequently retrieved at the axon initial segment (AIS) by a microtubule- and/or actin-based mechanism. Dynamic sorting along the PAEZ-AIS continuum can thus explain the polarized distribution of cytoplasmic organelles between the axonal and somatodendritic domains.

## Introduction

**Figure 1:**
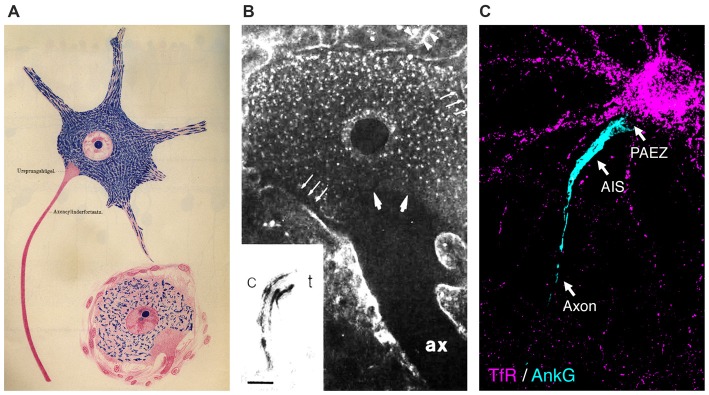
**Somatodendritic organelles are unable to enter the axon beginning at the level of the axon hillock. (A)** Illustration of spinal cord neurons showing exclusion of Nissl bodies (rough endoplasmic reticulum (ER)) from the proximal axon (taken from Held, 1895). **(B)** Exclusion of wheat germ agglutinin (WGA) staining from the axon hillock in a catfish giant electromotoneuron. ax, axon. Inset: WGA labels the *cis* (c)- to *trans* (t)-Golgi stacks; scale bar = 0.5 μm. Reprinted from Neuroscience, 52(3), Braun et al., “Cytoplasmic segregation and cytoskeletal organization in the electric catfish giant electromotoneuron with special reference to the axon hillock region”, 745-756, 1993 with permission from Elsevier. **(C)** Transferrin receptor (TfR)-containing somatodendritic vesicles (most of them classical early and recycling endosomes) (magenta) are excluded from the axon at the level of the pre-axonal exclusion zone (PAEZ), proximal to the axon initial segment (AIS; cyan) in cultured rat hippocampal neurons. Although these images were obtained using different methodologies and neuronal types, they all coincide in revealing a region of the axon hillock devoid of somatodendritic markers, which was recently defined as the PAEZ (Farías et al., 2015).

Among the first properties of neurons described by neuroanatomists was their polarization into distinct compartments—an axon, dendrites, and cell body or soma (Figure 1A; Deiters, 1865). As early as the first decade of the 20th century, Ramón y Cajal (1906) proposed that this structural polarity could explain the unidirectional transmission of information within and between neurons, a property he famously termed “dynamic polarization”. Although the intervening century has seen a revision of this one-way theory of neurotransmission, the notion of cellular structure as a critical determinant of function has become a core principle in biology.

Despite the enormous heterogeneity in neuronal form and function, a given neuron consists of a roughly spherical soma and one or more narrow membranous extensions, or neurites (Peters et al., 1991; Raine, 1999). Neurites are further subdivided into branched, tapering dendrites and a single thin axon, which may extend for long distances before branching into terminals (Peters et al., 1991; Raine, 1999). Although the soma and dendrites differ in shape, in terms of protein and organelle distribution the two are often treated as a unified somatodendritic domain. The somatodendritic and axonal domains may be differentiated by the presence of subdomains with unique roles in neurotransmission. The transmission of information between neurons most often occurs at synapses formed by juxtaposition of a postsynaptic site on the somatodendritic domain and a presynaptic axon terminal. The plasma membrane of postsynaptic terminals is enriched in neurotransmitter receptors underlain by a complex scaffold of structural and signaling proteins (Ziff, 1997; Collins et al., 2006; Lasiecka et al., 2008). In contrast, the plasma membrane of presynaptic terminals is characterized by neurotransmitter transporters, synaptic vesicle docking and fusion machinery, and a different submembranous matrix of structural proteins (Palay, 1956; Burns and Augustine, 1995; Ziv and Garner, 2004). Biosynthetic sorting of proteins to the somatodendritic and axonal plasma membrane domains involves packaging into distinct populations of transport carriers in the neuronal soma for subsequent delivery to their corresponding destinations. Transcytosis and local synthesis also contribute to the polarized distribution of some cargos (Horton and Ehlers, 2003; Lasiecka et al., 2008).

In addition to the plasma membrane, cytoplasmic organelles exhibit a polarized distribution in neurons (Figures 1B,C; Braun et al., 1993; Horton and Ehlers, 2003; Maday et al., 2014; Farías et al., 2015). For example, classical early endosomes, the rough endoplasmic reticulum (ER), and the Golgi complex are all largely prevented from entering the axon (Palay et al., 1968; Ramírez and Couve, 2011; Farías et al., 2015). The Golgi complex in particular has a characteristic distribution: in addition to the familiar juxtanuclear cisternae found in most cell types, small Golgi outposts are found near dendritic branch points, and even more substantial Golgi stacks are observed in larger dendrites (Hanus and Ehlers, 2008; Baas and Lin, 2011; Mikhaylova et al., 2016). Synaptic vesicles are mostly found in axon terminals, although synaptic vesicle precursors originate in the soma before migrating into the axon (Pigino et al., 2012). In contrast, the smooth ER, mitochondria, late endosomes, lysosomes, peroxisomes, autophagosomes, and dense-core vesicles are largely nonpolarized in distribution (Krijnse-Locker et al., 1995; Ligon and Steward, 2000; Farías et al., 2015; Lipka et al., 2016), although they may exhibit different properties within each domain (Overly et al., 1996).

## Fences and Borders: The Axon Initial Segment and Pre-Axonal Exclusion Zone

To achieve such polarized organization of the plasma membrane and cytoplasmic organelles, the neuron must selectively transport organelles to their intended destinations and, following transport, maintain their segregation to different domains. At the plasma membrane, the major boundary between the axonal and somatodendritic domains lies at the axon initial segment (AIS), a highly organized surface region of the proximal axon containing voltage-gated ion channels bound to an underlying assembly of ankyrin G (AnkG) and β-IV spectrin (Rasband, 2010). Dense clustering of Na^+^ and K^+^ channels within the AIS is critical for action potential initiation. The AIS also acts as a diffusion barrier for transmembrane proteins and lipids between the somatodendritic and axonal domains (Kobayashi et al., 1992; Winckler et al., 1999; Nakada et al., 2003; Rasband, 2010). The transmembrane proteins and associated lipids concentrated at the AIS have been proposed to act as a “picket fence” that obstructs passage of other transmembrane proteins and lipids between the two plasma membrane domains (Kusumi et al., 2012). Knockdown of AnkG in fully polarized neurons disassembles the AIS and results in loss of proximal axon identity, a phenomenon that may be at least partly due to the removal of the lateral diffusion barrier (Hedstrom et al., 2008; Sobotzik et al., 2009; Song et al., 2009; Jenkins et al., 2015).

In addition to its role in segregating plasma membrane proteins, the AIS has been proposed to function as a selective filter for cytoplasmic organelles (Song et al., 2009; Al-Bassam et al., 2012; Watanabe et al., 2012). A model developed in studies of vesicular transport carriers originating from the soma posits that carriers intended for the axon are freely able to pass through this filter, while somatodendritic carriers are blocked by an actin-dependent mechanism (Lewis et al., 2009; Song et al., 2009; Al-Bassam et al., 2012; Watanabe et al., 2012). In this model, the AIS functions either as a physical barrier that prevents axonal entry of somatodendritic carriers or as a scaffold for myosin Va-mediated retrieval of somatodendritic carriers that enter this segment. This view of the AIS as a sieve for cytoplasmic organelles, however, has been challenged by several findings. First, polarized organelle transport arises prior to AIS formation during neuronal development (Bradke and Dotti, 1997; Petersen et al., 2014; Farías et al., 2015). Moreover, axonal exclusion of somatodendritic carriers is achieved even when subsequent AIS formation is prevented by AnkG knockdown (Farías et al., 2015), and AnkG-null neurons maintain axonal identity as few as 50 μm from the soma (Jenkins et al., 2015). In terms of the proposed structure, super-resolution microscopy and platinum replica electron microscopy have shown that actin in the AIS exists as rings or sparse filaments, and not the dense or polarized structures required by the filter model (Xu et al., 2013; Jones et al., 2014; Leterrier et al., 2015). A key result cited in support of the AIS as an actin-based filter is the disruption of somatodendritic polarity when actin-depolymerizing drugs are applied to neurons (Lewis et al., 2009; Song et al., 2009). However, actin depolymerization has also been shown to cause missorting of somatodendritic proteins into axonal carriers at the Golgi complex (Petersen et al., 2014). Kuijpers et al. (2016) have recently proposed a different mechanism for the retrieval of somatodendritic vesicles that enter the AIS through AnkG-dependent recruitment of the proteins NDEL1 and LIS1, which activate the minus end-directed microtubule motor dynein on these vesicles for retrograde transport to the soma. Therefore, polarized organelle distribution can be achieved in the absence of the AIS, although myosin- and/or dynein-based retrieval mechanisms may fine-tune this distribution upon AIS assembly.

Although most studies to date have focused on the AIS as the boundary for somatodendritic and axonal organelles, a recent study has shown that in cultured hippocampal neurons most somatodendritic-specific organelles, such as somatodendritic carriers, the Golgi complex, and the rough ER, are prevented from entering the axon at a more proximal “pre-axonal exclusion zone” (PAEZ) present in the axon hillock or at the base of axons that emanate from dendrites (Farías et al., 2015; Figures 1A–C). This zone is defined at its proximal edge by a sharp decrease in the abundance of somatodendritic-specific organelles at the cytoplasmic transition from the perikaryon to the axon hillock. The distal border of the PAEZ occurs where the expression of AIS proteins begins, following the narrowing of the axon. Exclusion at the PAEZ applies specifically to organelles in the cytoplasm, as somatodendritic-specific proteins are found in the plasma membrane up to the AIS (Winckler et al., 1999; Farías et al., 2015). Further experiments showed that when somatodendritic transmembrane proteins are appended with a kinesin-1-binding peptide, vesicular carriers containing those proteins are able to traverse the PAEZ into the axon (Farías et al., 2015). Polarized sorting, then, may rely not on a filter-like exclusion of certain classes of organelles, but rather on the selective attachment of those organelles to appropriately targeted microtubule motors. These findings support an alternative model in which the main determinant of polarized transport is the ability of organelles to interact with specific microtubule motors that drive movement along different microtubule tracks (Braun et al., 1993; Nakata and Hirokawa, 2003; Jacobson et al., 2006; Konishi and Setou, 2009; Hammond et al., 2010; Nakata et al., 2011; Farías et al., 2015).

## Many Paths to Tread: The Polarized Neuronal Cytoskeleton

In building a model of polarized transport, it is important to consider the key ways in which neurons differ from other cells in their cytoskeletal architecture. Although actin and intermediate filaments are found throughout the cytoplasm, long-range organelle movement in neurons relies primarily on the microtubule cytoskeleton (Maday et al., 2014). Microtubules in mature neurons do not arise from a central organizing center (Horton and Ehlers, 2003; Kapitein and Hoogenraad, 2015), allowing the establishment of arrays with either uniform or mixed orientations and thus an additional form of cytoskeletal polarization across domains (Akhmanova and Hoogenraad, 2015; Yau et al., 2016).

Perhaps unsurprisingly, axonal and somatodendritic domains in neurons differ substantially in their microtubule arrangement (Kapitein and Hoogenraad, 2015; Yau et al., 2016). Although axonal microtubules are non-centrosomal, they are oriented uniformly, with more stable minus ends proximal to the nucleus and highly dynamic plus ends extending distally (Burton and Paige, 1981; Heidemann et al., 1981; Akhmanova and Hoogenraad, 2015). The minus end-binding protein CAMSAP2 is required for the stability of microtubule arrays throughout the neuron and is enriched at minus ends proximal to the AIS (Yau et al., 2014). In addition, the microtubule-associated protein (MAP) TRIM46 plays a critical role in organizing parallel microtubule bundles spanning the PAEZ and extending into the AIS (van Beuningen et al., 2015). During neuronal development, TRIM46 localizes to the one neurite destined to become the axon and promotes formation of microtubule bundles prior to either axon specification or AIS assembly (van Beuningen et al., 2015).

Dendrites exhibit less microtubule polarity, with a roughly balanced mixture of plus end-out and minus end-out microtubules (Baas et al., 1988; Yau et al., 2016). Early studies reported an increasing proportion of plus end-out microtubules toward the distal end of the dendrite (Baas et al., 1988, 1989); however, a recent analysis has challenged this finding, suggesting that orientations are equally mixed throughout the dendrite (Yau et al., 2016).

## The Long Walk: Microtubule Motors and Polarized Transport

Long-range organelle movement in neurons is dominated by the action of microtubule-based motors (Maday et al., 2014). Numerous kinesin families exist and are subdivided on the basis of structure and directionality of movement. The majority of kinesins possess N-terminal motor domains and walk toward microtubule plus ends, while a few with C-terminal motor domains move toward minus ends (Hirokawa et al., 2009). A given kinesin family contains one or more genes encoding kinesin heavy chains (KIFs), which may interact with a variety of adaptors (Hirokawa et al., 2009). The main minus end-directed microtubule motor in neurons is, however, a structurally distinct protein, dynein (Kapitein et al., 2010; Maday et al., 2014). Given the uniform orientation of axonal microtubules, most kinesins with N-terminal motor domains drive anterograde transport in the axon, while dynein mediates retrograde axonal transport (Maday et al., 2014). In dendrites, a given motor may move anterogradely or retrogradely with respect to the soma, depending on the orientation of the microtubule to which it binds. A relay mechanism involving sequential interactions with different kinesins and dynein may be required for transport to distal regions of dendrites (Welte et al., 1998; Levi et al., 2006).

A number of features of kinesin movement have been characterized. Certain plus end-directed kinesins move only into the axon, including members of the kinesin-1 family, which mediate axonal transport of synaptic vesicle precursors, carriers for plasma membrane proteins, and mitochondria (Jacobson et al., 2006; DeBoer et al., 2008; Hirokawa et al., 2009; Huang and Banker, 2012; Maday et al., 2014). Other kinesins, such as members of the kinesin-3 family, can drive transport of early endosomes and carriers for various presynaptic proteins into dendrites (Hirokawa et al., 2009; Huang and Banker, 2012; Farkhondeh et al., 2015; Lipka et al., 2016), where they may move bidirectionally, given the mixed orientation of dendritic microtubules (Baas et al., 1989; Yau et al., 2016). Of great interest in the study of neuronal polarity is the preferential binding of kinesins to unique microtubule populations and thus the determination of domain specificity for a given motor. A useful technique in locating initial kinesin binding sites has been the expression of “rigor” kinesin mutants, which bind microtubules without walking along them (Nakata and Hirokawa, 1995, 2003). For example, rigor mutants of the kinesin-1 family members KIF5A and KIF5B preferentially localize to microtubule bundles spanning the PAEZ, suggesting that a unique feature of these bundles favors binding of KIF5 and thus allows polarized transport into the axon (Nakata and Hirokawa, 2003; Farías et al., 2015).

The ability of a specific kinesin to recognize and bind a unique subset of microtubules may depend on MAPs or posttranslational modifications (PTMs) of tubulin (Nakata and Hirokawa, 2003; Jacobson et al., 2006; Hammond et al., 2010; Nakata et al., 2011; Farías et al., 2015). Such preferences can be domain-specific; for example, binding of the MAP DCLK1 to dendritic microtubules is required for dendritic dense-core vesicle trafficking mediated by KIF1A-C, members of the kinesin-3 family (Lipka et al., 2016). Binding of KIF5A, on the other hand, occurs preferentially along GTP- and acetylated tubulin-rich microtubule bundles spanning the PAEZ, and overexpression of an acetylation-mimic tubulin mutant or the hMB11 intrabody to GTP-tubulin disrupts the selective binding of KIF5 to axonal microtubules (Nakata et al., 2011; Farías et al., 2015). PTMs implicated in polarized movement into the axon may provide an upstream mechanism for recruiting specific motors, and by extension their associated cargos, to the axon (Hammond et al., 2010). A broader “MAP-PTM-kinesin code” is speculated to regulate kinesin binding to microtubule subpopulations (Liu et al., 2012; Atherton et al., 2013), although the diversity of proteins involved leaves large numbers of potential interactions yet to be tested.

Cargos themselves also modulate kinesin movement and can, in effect, steer motors into a particular domain (Setou et al., 2002). Certain kinesin-organelle associations are direct, through interactions between kinesin tail domains and transmembrane cargos or membrane lipids (Hirokawa et al., 2009). Other interactions require adaptor and scaffold proteins, for example the various kinesin light chains (KLCs) (Gyoeva et al., 2004). KLCs display both cargo and motor specificity, and isoforms targeting heavy chains to the Golgi, mitochondria, and other organelles have been identified (Khodjakov et al., 1998; Gyoeva et al., 2000). Beyond the KLCs, a large assortment of adaptors, GTPases and their effectors, and other regulatory proteins controls the binding, movement, and unloading of cargo (Hirokawa et al., 2009; Maday et al., 2014). In a microtubule motor-based view of neuronal polarization, all of these proteins work in concert to determine the distribution of organelles between the axonal and somatodendritic domains.

## Outlook: A Transport-Focused View of Polarized Organelle Distribution in Neurons

**Figure 2:**
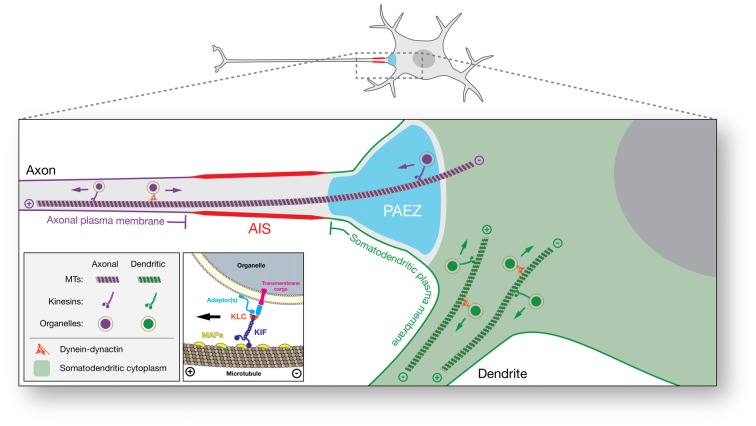
**Schematic representation of microtubule motor-based polarized distribution of organelles in neurons.** In this model, the ability of an organelle to move along microtubule tracks to the axonal or somatodendritic domain is determined by the microtubule motors to which it binds. Organelles that bind axonally-directed kinesins are capable of crossing the PAEZ and moving into the axon. On the other hand, organelles that bind dendritically-directed kinesins or dynein do not traverse the PAEZ and are instead directed to the dendrites. A fraction of somatodendritic organelles that escape sorting at the PAEZ can be retrieved by dynein- or myosin-dependent retrograde transport at the AIS. Inset: detailed view of interactions mediating plus end-directed organelle transport. Adaptor or scaffold proteins determine the binding of organelles to specific microtubule motors. Shown are generic adaptors mediating interactions of kinesin light chain (KLC) with a transmembrane cargo or the organelle membrane. Some kinesin heavy chains (KIFs) interact directly with transmembrane cargos or organelle membranes without the need for adaptors. Microtubule-associated proteins (MAPs) and posttranslational modifications (PTMs) of tubulin are asymmetrically distributed in neurons and regulate the binding of specific kinesins to subpopulations of axonal and somatodendritic microtubules. While segregation of axonal and somatodendritic organelles is mostly established at the level of the PAEZ, separation of axonal and somatodendritic plasma membrane proteins and lipids occurs at the surface of the AIS.

In conclusion, a growing body of evidence supports a model in which the main determinants of polarized organelle distribution in neurons are differential interactions of cytoplasmic organelles with various cargo adaptors, microtubule motors, and microtubule tracks (Figure 2). At its simplest, this model propounds that binding of organelles to axonally-directed kinesins drives transport to the axon, whereas binding to dendritically-directed kinesins and/or dynein promotes movement within the soma and into the dendrites. The exact combinations of factors that contribute to these differential interactions, however, remain to be fully elucidated for most organelles. This divergence is particularly manifest at the PAEZ, highlighting this region as the cytoplasmic boundary between the axonal and somatodendritic domains (Farías et al., 2015). Interestingly, this same boundary was apparent in previous morphological studies of different neuronal types, some dating back to the 19th century (Figures 1A,B; Held, 1895; Braun et al., 1993). While most studies to date have focused on vesicular transport carriers, we speculate that other organelles such as classical early endosomes, the Golgi complex, and the rough ER are also excluded from the PAEZ and the axon by their failure to associate with axonal kinesins and/or their early association with dendritic kinesins or dynein. For some of these organelles, stable anchoring to other cytoskeletal structures may also prevent unwanted transport into the axon (Gurel et al., 2014). This model is compatible with the AIS playing a complementary role by supporting the retrieval of somatodendritic organelles that escape sorting at the PAEZ. This role of the AIS could depend on the same microtubule motor-based mechanism that operates at the PAEZ (Farías et al., 2015), and/or the AIS-specific recruitment of dynein to the escaped somatodendritic organelles (Kuijpers et al., 2016). Myosin motors could also contribute to this retrieval through association with actin structures tethered to the submembranous AIS scaffold (Lewis et al., 2009; Al-Bassam et al., 2012; Watanabe et al., 2012). Dynamic sorting along the PAEZ-AIS continuum may thus be sufficient to determine the polarized distribution of cytoplasmic organelles between the somatodendritic and axonal domains in the absence of a physical filter.

The understanding of polarized organelle transport is still evolving, and the findings discussed here warrant more detailed structural, biochemical, and imaging studies of organelle dynamics in and around the proximal axon. At a broader scope, a fuller description of neuronal polarity will require the coupling of these findings to knowledge of the pathways regulating expression of adaptors, motors, and the machinery for tubulin modifications. Given the relevance of neuronal polarity to human development and disease, further work should also evaluate this model in the context of the multiple modes of polarization exhibited by neurons, particularly in higher-order systems such as tissues and whole organisms (Namba et al., 2015).

## Author Contributions

DJB prepared the preliminary draft of the manuscript. All authors (DJB, GGF, CMG, JSB) contributed to the further writing and revision of the manuscript and the associated review of the literature.

## Conflict of Interest Statement

The authors declare that the research was conducted in the absence of any commercial or financial relationships that could be construed as a potential conflict of interest.
